# Effect of tumor-infiltrating lymphocytes depending on the presence of postmastectomy radiotherapy on the prognosis in pT1-2N1M0 breast cancer

**DOI:** 10.3389/fonc.2023.1175965

**Published:** 2023-08-04

**Authors:** Lina Zhang, Tiantian Tang, Lei Liu, Chunxiao Li, Yuntao Li, Cuizhi Geng

**Affiliations:** Breast Center, The Fourth Hospital of Hebei Medical University, Shijiazhuang, China

**Keywords:** tumor-infiltrating lymphocytes, breast cancer, postmastectomy radiotherapy, prognosis, local recurrence

## Abstract

**Background:**

Currently, it remains unclear regarding the association between tumor-infiltrating lymphocytes (TILs) and the efficacy of postoperative radiotherapy in primary tumors. Here we attempted to investigate the effect of TILs depending on the presence of postmastectomy radiotherapy (PMRT) on the prognosis in pT1-2N1M0 breast cancer.

**Methods:**

The clinical data of pT1-2N1M0 breast cancer patients undergoing mastectomy and axillary lymph node dissection were retrospectively analyzed. The effect of TILs on the prognosis was assessed based on the infiltration degree (low: TILs ≤10%, high: TILs >10%), and then the prognosis of patients with low and high infiltration of TILs was analyzed based on presence or absence of PMRT.

**Results:**

Totally 213 patients were eligible for the study, including 162 cases of low infiltration and 51 of high infiltration. High-infiltration patients tended to be ER/PR-negative, HER2-positive, and have high histological grade. The infiltration in triple-negative and HER2-positive subtypes was higher compared with Luminal A subtype. Regarding local-regional recurrence-free survival, recurrence-free survival, and overall survival (OS) rates, the differences were all inapparent whether in high- and low-infiltration patients or in high-infiltration patients with/without PMRT. Compared with those without PMRT, low-infiltration patients with PMRT showed a significantly increased OS rate (92.8% *vs.* 80.0%, *p*=0.023). Multivariate analysis further confirmed PMRT as an independent predicator of OS in low-infiltration patients (HR: 0.228, 95%CI: 0.081-0.644, *p*=0.005).

**Conclusion:**

High infiltration of TILs in pT1-2N1M0 breast cancer may be associated with clinicopathological factors. Low-infiltration patients, but not high-infiltration patients, may derive survival benefits from PMRT.

## 1 Introduction

Carcinogenesis is associated with dysfunction of immune cells in the body (1). The immunity of patients with malignant tumors is usually low, which makes immune cells unable to recognize and kill the tumor cells. Tumor microenvironment (TME) comprising the cells around the tumor and non-cellular components plays a crucial role in tumor growth, migration, and metastasis. Tumor-infiltrating lymphocytes (TILs), an important component of the TME, are composed of immune cells infiltrating the tumor cells and have been identified in a variety of solid tumors, including breast cancer, colon cancer, cervical cancer, lung cancer and melanoma (2, 3). Previous studies have demonstrated that the TILs in breast cancer tissue are mainly composed of cytotoxic T (CD8^+^) cells, helper T (CD4^+^) cells, B (CD19^+^) cells and natural killer (NK) cells (4, 5), and the subtypes of TILs can affect the tumor cells and immune cells in multiple ways, consequently contributing to either an anti-tumor or pro-tumor effect (6–8).

There are studies suggesting that TILs are associated with the prognosis of patients with early-stage triple negative breast cancer (TNBC), the higher lymphocytic infiltration, the better the prognosis (9–11). However, this association is not identified in estrogen receptor (ER)-positive/human epidermal growth factor receptor 2 (HER2)-negative and HER2-positive breast cancers (11). In breast cancer patients treated with neoadjuvant chemotherapy, TILs are linked to the pathological complete response (pCR) and long-term prognosis (12–15). Additionally, TILs are predictive of the response to anthracycline- and platinum-based chemotherapy drugs, as well as anti-HER2 agent trastuzumab (10, 11, 15).

Radiotherapy, one of the important adjuvant therapies for breast cancer, can eliminate the dormant residual lesions to reduce the risk of local recurrence (16). It can not only result in cell necrosis and apoptosis by damaging the double-strand DNA (17, 18), but also can affect the tumorigenesis and progression by modulating the immune system and TME (19, 20). The evidence showed that the tumor necrosis induced by local radiotherapy could activate the immune system, leading to tumor shrinkage (21–23). However, this pathogenesis is still undefined. Regarding the association of TILs with the effect of postoperative radiotherapy on ipsilateral breast tumor recurrence, Kovacs et al. demonstrated that breast cancer patients with high TILs had a lower risk of local recurrence after breast-conserving surgery, and those with low TILs might derive larger benefits from radiotherapy (24). Tramm et al. found that high TILs were conductive to predicting the improvement of overall survival (OS) from postmastectomy radiotherapy (PMRT) in breast cancer, which was very significant in ER- tumors (25). Notably, there are still lack of studies on the association between TILs and the effect of postoperative radiotherapy in primary tumors.

For pT1-2N1M0 breast cancer patients, it remains controversial about whether PMRT should be performed. The current guidelines, such as National Comprehensive Cancer Network guidelines, have offered that for N1 breast cancer patients, clinical or pathological high-risk factors should be considered to determine whether PMRT is performed (26). However, in clinical practice, there is lack of unified standards for determination of clinical or pathological high-risk factors due to complexity, leading to frequently occurring biases from different physicians in radiotherapy decision-making of pT1-2N1M0 patients. Hence, in the present study, we attempted to investigate the effect of TILs depending on the presence of PMRT on the prognosis of pT1-2N1M0 breast cancer patients, with the aim of providing more evidence for clinical decision-making.

## 2 Materials and methods

### 2.1 Study population

The pT1-2N1M0 breast cancer patients who underwent mastectomy and axillary lymph node dissection (ALND) in The Fourth Hospital of Hebei Medical University between March 2011 and December 2015 were enrolled into the study. Inclusion criteria included (1): age ≥18 years; (2) primary invasive breast cancer initially diagnosed with pT1-2N1M0. According to pathological results, the largest diameter of the primary invasive lesion was 5 cm at most, and there were 1-3 axillary lymph node metastases but no distant metastases; (3) patients undergoing mastectomy combined with ALND, but not receiving any neoadjuvant therapies like chemotherapy, endocrine therapy, targeted therapy, or radiotherapy; (4) patients without the history of any other malignant tumors, including previous history of breast cancer. Those with insufficient clinicopathological information and follow-up data were excluded.

Written informed consent was obtained from each patient. This study was approved by the Institutional Review Board of The Fourth Hospital of Hebei Medical University (approval No.: 2020115) and was performed in accordance with the principles of Declaration of Helsinki and local regulations.

### 2.2 Collection of clinical data

The clinical data of patients enrolled in this study were extracted through consultation of the electronic medical record, involving age, histological grade, tumor stage, number of axillary lymph nodes, ER, progesterone receptor (PR) and HER2 status, molecular subtype, as well as presence or absence of lymphovascular invasion (LVI).

### 2.3 Evaluation of TILs

According to the evaluation criteria of TILs in breast cancer recommended by an International TILs Working Group 2014, TILs are divided into intratumoral and stromal types. Intratumoral TILs are defined as the lymphocytes in tumor nests with cell-to-cell contact but no intervention of stroma, as well as directly interacting with the tumor cells. Stromal TILs are defined as the lymphocytes dispersed in the stroma that do not directly contact the tumor cells, which is recommended as the optimal parameter by an International TILs Working Group because the growth pattern of tumor cells may affect the distribution of lymphocytes in tumor nests, leading to a higher heterogeneity and fewer numbers (26).

**Figure 1:**
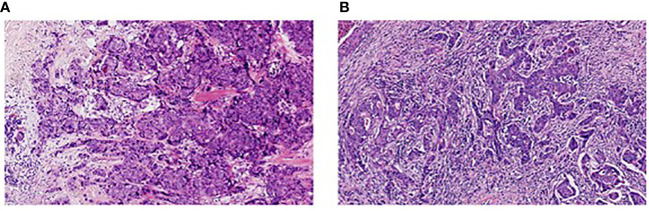
Infiltration of stromal tumor-infiltrating lymphocytes in breast cancer (H&E, 200×). **(A)** Low infiltration; **(B)** High infiltration.

In this study, TILs were assessed based on the evaluation criteria of TILs recommended by an International TILs Working Group 2014 (27). Two pathologists were responsible for reviewing hematoxylin and eosin (H&E)-stained tumor sections of all patients and interpreting the infiltration degree of stromal TILs. The mean value assessed by two pathologists was considered as the final score. As shown in **Figure 1**, TILs ≤10% were defined as low infiltration (**Figure 1A**), while TILs >10% were considered as high infiltration (**Figure 1B**).

### 2.4 Follow-up

All the patients were followed-up after mastectomy through further consultations and telephones, and the follow-up deadline was February 2020. The effect of TILs on the patients’ prognosis was first assessed based on the infiltration degree. Then, according to presence or absence of PMRT, the prognosis of patients with low and high infiltration of TILs was analyzed.

The primary observational endpoints included local-regional recurrence-free survival (LRFS), recurrence-free survival (RFS) and OS. LRFS was defined as the duration of time from surgery until any recurrence of ipsilateral chest, breast, regional lymph node recurrence, or death occurred from any cause. RFS was defined as the duration of time from surgery until any recurrence of ipsilateral chest, breast, regional lymph node recurrence, distant metastases, or death occurred from any cause. OS was defined as the time from surgery to death from any cause or the last date of contact for a surviving patient.

### 2.5 Statistical analysis

All the data were analyzed using SPSS 24.0 statistical software (SPSS Inc., Chicago, IL, USA). χ^2^ or Fisher’s exact test was utilized to analyze the categorical data, expressing as the case number and percentage [n(%)]. Survival curves were drawn using the Kaplan-Meier method and compared by Log-rank test. Univariate and multivariate Cox proportional-hazards models were employed to determine the influencing factors for the prognosis, and hazards ratios (HR) and 95% confidence interval (CI) were calculated, respectively. All statistical tests were two-sided. The value of *p*<0.05 was considered statistically significant.

## 3 Results

### 3.1 Patient characteristics

From March 2011 to December 2015, a total of 213 cases were eligible for the study and enrolled into the analysis, with the mean infiltration value of 6% in TILs (range: 3%, 10%), among whom 162 cases were assessed to have low infiltration of TILs (≤10%) and 51 had high infiltration (>10%) of TILs. The baseline characteristics of patients with high and low infiltration of TILs were compared in **Table 1**. It could be observed that by comparison to those with low infiltration, patients with high infiltration of TILs tended to be ER/PR-negative (49.0% *vs.* 16%, *p*=0.000), HER2-positive (41.2% *vs.* 21.0%, *p*=0.004), and grade III (66.7% *vs.* 33.3%, *p*<0.001). No significant differences were shown in age, tumor stage and number of axillary lymph nodes (*p*>0.05).

**Table 1:** Relationship between TILs infiltration in breast cancer tissue and clinicopathological characteristics, n(%).

Characteristics	TILs		*p*
	Low infiltration (n=162)	High infiltration (n=51)	
Age, years			0.952
≤40	28(17.3)	9(17.6)	
>40	134(82.7)	42(82.4)	
Histological grade			<0.001
I-II	105(64.8)	17(33.3)	
III	57(35.2)	34(66.7)	
Tumor stage			0.184
T1	71(43.8)	17(33.3)	
T2	91(56.2)	34(66.7)	
Number of axillary lymph nodes			0.903
1	81(50.0)	26(51.0)	
2-3	81(50.0)	25(49.0)	
ER/PR status			<0.001
ER/PR positive	136(84.0)	26(51.0)	
Both negative	26(16.0)	25(49.0)	
HER2 status			0.004
Positive	34(21.0)	21(41.2)	
Negative	128(79.0)	30(58.8)	
LVI			0.400
Yes	78(48.1)	28(54.9)	
No	84(51.9)	23(45.1)	
Molecular subtypes			<0.001
Luminal A	50(30.9)	4(7.8)	0.002^a^
Luminal B	87(53.7)	25(49.0)	0.001^b^
TNBC	16(9.9)	10(19.6)	
HER2 positive	9(5.5)	12(23.6)	<0.001^c^
Clinical risk model*			0.071
Low-risk	42(25.9)	7(13.7)	
High-risk	120(74.1)	44(86.3)	

“a, b, and c” represent Luminal A versus TNBC, Luminal A versus HER2 positive, and Luminal B versus HER2 positive, respectively. “*” represents the tumor samples of patients are detected and assessed by RecurIndex assay.

TILs, tumor-infiltrating lymphocytes; ER, estrogen receptor; PR, progesterone receptor; HER2, human epidermal growth factor receptor 2; LVI, lymphovascular invasion; TNBC, triple negative breast cancer.

According to St. Gallen consensus for molecular classification of breast cancer 2013 (26), breast cancer was classified into Luminal A subtype, Luminal B subtype, TNBC and HER2-positive subtype. In terms of different subtypes, the difference was pronounced between the patients with low and high infiltration of TILs (*p*<0.001). Additionally, the infiltration in triple-negative and HER2-positive subtypes was higher compared with Luminal A subtype (*p*=0.002, *p*<0.001), and the infiltration of TILs in HER2-positive breast cancer was significantly higher than that in Luminal B subtype (*p*=0.001; **Table 1**).

### 3.2 Association of TILs infiltration with prognosis

**Figure 2:**
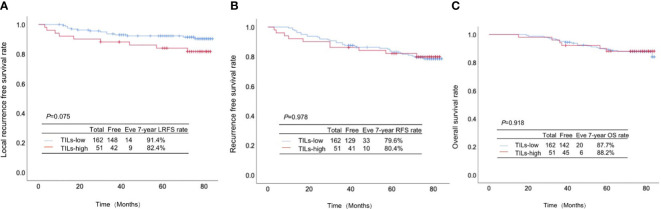
Association between TILs infiltration and the prognosis in pT1-2N1M0 breast cancer. **(A)** Local-regional recurrence-free survival rate, **(B)** recurrence-free survival rate and **(C)** overall survival rate between the patients with high and low infiltration of TILs.

Postoperatively, all the patients were followed up for 7 years. Among 162 patients with low infiltration of TILs, 14, 33 and 20 cases experienced local-regional recurrence (LRR), recurrence and death, respectively, while in 51 patients with high infiltration of TILs there were 9 cases with LRR, 10 cases with recurrence and 6 deaths. The differences were all inapparent between the patients with high and low infiltration of TILs regarding the LRFS rate (91.4% *vs.* 82.4%, *p*=0.075; **Figure 2A**), RFS rate (79.6% *vs.* 80.4%, *p*=0.978; **Figure 2B**) and OS rate (87.7% *vs.* 88.2%, *p*=0.918; **Figure 2C**).

### 3.3 PMRT-based subgroup analysis

**Figure 3:**
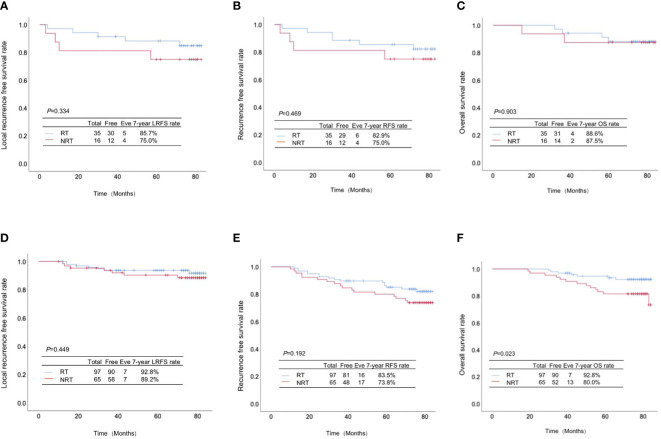
Association of TILs infiltration with the effect of postmastectomy radiotherapy (PMRT) on the prognosis of pT1-2N1M0 breast cancer patients. **(A)** Local-regional recurrence-free survival rate, **(B)** recurrence-free survival rate and **(C)** overall survival rate between the high-infiltration patients with and without PMRT; **(D)** Local-regional recurrence-free survival rate, **(E)** recurrence-free survival rate and **(F)** overall survival rate between the low-infiltration patients with and without PMRT.

Among 51 patients with high infiltration of TILs, 35 cases underwent PMRT, while 16 didn’t. There were no significant differences between the high-infiltration patients with and without PMRT in LRFS rate (85.7% *vs.* 75.0%, *p*=0.334, **Figure 3A**), RFS rate (82.9% *vs.* 75.0%, *p*=0.469, **Figure 3B**), and OS rate (88.6% *vs.* 87.5%, *p*=0.903, **Figure 3C**).

Totally 97 cases out of 162 patients with low infiltration of TILs received PMRT, while 65 didn’t. Despite absence of significant differences in LRFS rate (92.8% *vs.* 89.2%, *p*=0.449, **Figure 3D**) and RFS rate (83.5% *vs.* 73.8%, *p*=0.192, **Figure 3E**), the OS rate of low-infiltration patients receiving PMRT was significantly higher than those without PMRT (92.8% *vs.* 80.0%, *p*=0.023, **Figure 3F**).

Univariate and multivariate Cox models of OS in 162 patients with low infiltration of TILs were established in **Table 2**. It could be observed that PMRT was an independent predicator for OS in patients with low infiltration of TILs whether through univariate analysis (HR: 0.361, 95%CI: 0.144-0.904, *p*=0.030) or through multivariate analysis (HR: 0.228, 95%CI: 0.081-0.644, *p*=0.005).

**Table 2:** Univariate and multivariate Cox models of OS in patients with low infiltration of TILs.

Variables	Univariable		Multivariable	
	HR (95%CI)	*p* value	HR (95%CI)	*p* value
Chemotherapy				
No	1.000 (reference)		1.000 (reference)	
Yes	1.203 (0.351-4.120)	0.769	1.565 (0.426-5.749)	0.500
Radiotherapy				
Non-PMRT	1.000 (reference)		1.000 (reference)	
PMRT	0.361 (0.144-0.904)	0.030	0.228 (0.081-0.644)	0.005
Age, years				
≤40	1.000 (reference)		1.000 (reference)	
>40	0.770 (0.257-2.306)	0.641	0.627 (0.185-2.125)	0.454
ER/PR status				
Negative	1.000 (reference)		1.000 (reference)	
Positive	1.056 (0.309-3.605)	0.931	1.759 (0.455-6.799)	0.413
HER2 status				
Negative	1.000 (reference)		1.000 (reference)	
Positive	2.220 (0.883-5.578)	0.090	2.125 (0.770-5.866)	0.146
LVI				
No	1.000 (reference)		1.000 (reference)	
Yes	0.939 (0.389-2.268)	0.889	0.952 (0.381-2.376)	0.915
Number of axillary lymph nodes				
1	1.000 (reference)		1.000 (reference)	
2-3	1.599 (0.653-3.913)	0.304	2.098 (0.766-5.748)	0.149
Tumor stage				
T1	1.000 (reference)		1.000 (reference)	
T2	2.499 (0.908-6.878)	0.076	2.172 (0.727-6.483)	0.165
Ki67, %				
≤20	1.000 (reference)		1.000 (reference)	
>20	2.947 (0.984-8.826)	0.054	1.988 (0.596-6.623)	0.263
Grade				
I-II	1.000 (reference)		1.000 (reference)	
III	1.868 (0.776-4.495)	0.163	1.254 (0.473-3.324)	0.649

TILs, tumor-infiltrating lymphocytes; OS, overall survival; ER, estrogen receptor; PR, progesterone receptor; HER2, human epidermal growth factor receptor 2; LVI, lymphovascular invasion; PMRT, postmastectomy radiotherapy; HR, hazard ratio; CI, confidence interval.

## 4 Discussion

Cytotoxic therapies including chemotherapy and radiotherapy can activate the body’s immune system (28–30). As a component of the TME, TILs are important immune predictors in the process of tumorigenesis and progression. In breast cancer with specific molecular subtypes, TILs can not only predict the prognosis and response to neoadjuvant chemotherapy, but also can predict the efficacy of anthracycline- and platinum-based chemotherapy drugs. However, it remains conflicting about the relationship between TILs and the effect of PMRT on the prognosis in pT1-2N1M0 breast cancer. In the present study, we demonstrated that patients with low infiltration of TILs may derive survival benefits from PMRT, with a lower risk of death compared with those not receiving PMRT, while for patients with high infiltration of TILs this positive effect of PMRT on the prognosis was not identified. Interestingly, Kovacs et al. also exhibited radiotherapy could decrease the risk of LRR and any recurrence in patients with low infiltration of TILs after breast-conserving surgery (24). Although our study did not confirm the association of PMRT with the risk of LRR and any recurrence in patients with low infiltration of TILs, our findings suggested that low TIL infiltration might be associated with sensitivity to PMRT, and patients with low infiltration of TILs were more likely to benefit from PMRT.

Currently, the relationship between TILs and the effect of radiotherapy is not fully understood. Our results indicated that the effect of radiotherapy was better in patients with low infiltration of TILs, which might be associated with a higher proportion of ER-positive patients who usually showed a better prognosis due to less invasion than ER-negative patients. A previous study has demonstrated that radiotherapy is more effective for breast cancers with less invasion, especially for Luminal-type (ER positive) breast cancer, and radiotherapy can decrease the cumulative incidence of ipsilateral breast cancer recurrence (31). ER can induce the production of reactive oxygen species, leading to genomic instability. It has a synergistic effect with radiotherapy on DNA damage of cancer cells to increase the effect of radiotherapy (32). Additionally, the impact of ER on cell cycles can also enhance the effect of radiotherapy on DNA damage (33, 34). Importantly, the benefits of patients with low infiltration of TILs from radiotherapy are more attributable to the changes in immune microenvironment caused by radiotherapy. Several studies have suggested that radiotherapy can induce the variations in the body’s immune cells and microenvironment and activate the anti-tumor effect of immune system (35–37).

Previous studies indicated the higher the TILs infiltration, the better the prognosis of TNBC patients (10, 11). In our study, however, all the differences were indistinctive in the LRFS rate, RFS rate and OS rate whether in high- or low-infiltration patients, which might be attributed to a higher proportion of patients with luminal-type breast cancer. Among 213 cases of pT1-2N1M0 breast cancer, 77.9% of patients were luminal-type breast cancer, while only 12.2% were TNBC. Additionally, pT1-2N1M0 breast cancer patients included in this study had similar TNM staging, leading to an analogical prognosis to some extent. Notably, patients with high infiltration of TILs tended to be ER/PR-negative, HER2 positive, and have high histological grade, and the infiltration in triple-negative and HER2-positive subtypes was higher than Luminal A subtype, which were supported by the previous research results (10, 11). Compared with the luminal subtype, the infiltration degree of TILs in triple-negative and HER2-positive subtypes was higher, but the prognosis was worse, which may lead to the presence of multiple unfavorable prognostic factors in patients with high infiltration of TILs. Hence, all these findings suggest that high infiltration of TILs may predict poor oncological characteristics. Notably, we observed that PMRT could improve the OS of patients with low infiltration of TILs, but not RFS and LRFS. However, in clinical practice the long-term benefits caused by PMRT are often transformed from local benefits. This may be related to the small sample size. In subsequent studies, we will further expand the sample size to explore the impact of TILs on the prognosis of this population and its possible mechanisms.

A major strength of this study was that it first investigated the effect of TILs depending on the presence of PMRT on the prognosis in pT1-2N1M0 breast cancer. For pT1-2N1M0 breast cancer, clinicians often make decisions on adjuvant radiotherapy based on whether the patient is combined with other clinical risk factors. Our results unveil that TILs may serve as a potential indicator for implementation of adjuvant radiotherapy in breast cancer, especially in the luminal-type breast cancer. Nevertheless, it was a retrospective study with the small sample size, and the association of PMRT with the risk of LRR and any recurrence was not identified whether in high- or low-infiltration patients. Additionally, the classification of luminal and non-luminal breast cancers was not included in univariate and multivariate COX analyses due to a great overlap between luminal and HR-positive breast cancers. In the future, we will continue to conduct more large-scale, prospective studies to validate our findings, hoping to find the key indicators that can accurately predict radiotherapy sensitivity and efficacy to aid in clinical decision-making.

In conclusion, high infiltration of TILs in pT1-2N1M0 breast cancer may be associated with clinicopathological factors, such as negative ER/PR, positive HER2 status, and high histological grade. Patients with low infiltration of TILs may derive survival benefits from PMRT, an independent predictor of OS, while these survival benefits are absent in patients with high infiltration of TILs.

## Data availability statement

The original contributions presented in the study are included in the article/supplementary material. Further inquiries can be directed to the corresponding author.

## Ethics statement

Written informed consent was obtained from each patient. This study was approved by the Institutional Review Board of The Fourth Hospital of Hebei Medical University (approval No.: 2020115) and was performed in accordance with the principles of Declaration of Helsinki and local regulations.

## Author contributions

LZ: Conceptualization, and writing-original draft, TT and LL: Methodology, and formal analysis, CL and YL: Investigation, data curation and formal analysis, CG: Conceptualization, writing-reviewing, supervision. All authors contributed to the article and approved the submitted version.
